# Fiber Optic Distributed Sensing Network for Shape Sensing-Assisted Epidural Needle Guidance

**DOI:** 10.3390/bios11110446

**Published:** 2021-11-11

**Authors:** Aida Amantayeva, Nargiz Adilzhanova, Aizhan Issatayeva, Wilfried Blanc, Carlo Molardi, Daniele Tosi

**Affiliations:** 1 School of Engineering and Digital Sciences, Nazarbayev University, Nur-Sultan 010000, Kazakhstan; aida.amantayeva@alumni.nu.edu.kz (A.A.); nargiz.adilzhanova@alumni.nu.edu.kz (N.A.); carlo.molardi@nu.edu.kz (C.M.); 2 Department of Engineering and Architecture, University of Parma, Parco Area delle Scienze 181/A, I-43124 Parma, Italy; aizhan.issatayeva@alumni.nu.edu.kz; 3 Université Côte d’Azur, INPHYNI, CNRS UMR7010, Avenue Joseph Vallot, 06108 Nice, France; Wilfried.Blanc@unice.fr; 4 National Laboratory Astana, Laboratory of Biosensors and Bioinstruments, Nur-Sultan 010000, Kazakhstan

**Keywords:** fiber-optic shape sensors, optical fiber sensor, distributed sensors, epidural anesthesia, epidural needle, smart surgical instruments

## Abstract

Epidural anesthesia is a pain management process that requires the insertion of a miniature needle through the epidural space located within lumbar vertebrae. The use of a guidance system for manual insertion can reduce failure rates and provide increased efficiency in the process. In this work, we present and experimentally assess a guidance system based on a network of fiber optic distributed sensors. The fibers are mounted externally to the needle, without blocking its inner channel, and through a strain-to-shape detection method reconstruct the silhouette of the epidural device in real time (1 s). We experimentally assessed the shape sensing methods over 25 experiments performed in a phantom, and we observed that the sensing system correctly identified bending patterns typical in epidural insertions, characterized by the different stiffness of the tissues. By studying metrics related to the curvatures and their temporal changes, we provide identifiers that can potentially serve for the (in)correct identification of the epidural space, and support the operator through the insertion process by recognizing the bending patterns.

## 1. Introduction

In recent years, the availability of low-cost miniaturized sensors has progressively allowed the realization of smart surgical instruments and needles [1] that combine the detection of biophysical parameters with therapeutic functions such as the delivery of anesthetic fluid [2], radiating a surrounding tissue with focused electromagnetic waves to deliver cancer therapies [3], or performing diagnostic tasks through endoscopic and photoacoustic imaging [4]. Multifunctional surgical devices benefit from having advanced and miniaturized on-board sensors that can either perform or contribute to diagnostic tasks or provide in situ detection of additional parameters to improve efficacy.

Electrical physical sensors have been used on-board some percutaneous or endoscopic devices, allowing substantial advantages in instruments that have a suitable form factor. Among others, micro-electromechanical pressure sensors have been substantially used in urology for the detection of bladder obstruction-related diseases [5] by packaging two miniature pressure sensors (with external hardware) into the bladder and into the rectal catheter. On the other hand, micro-thermocouples are currently featured within percutaneous radiofrequency and microwave applicators for thermal ablation [6], controlling the thermal dose delivered in the tissue.

In recent years, biomedical fiber optic sensors (FOS) have progressively improved the performance and the sensing methods for building the next generation of smart medical devices [7]. In this regard, FOS have two advantages over their electrical counterparts: (1) Fibers have a miniature form factor, which allows positioning several sensors within medical devices with efficient configurations that allow even stacking of an entire fiber bundle [4], and have semi-rigid packaging as in sensory cilia [8]. (2) While electrical sensors are inherently single-point sensors, optical fibers can have a plurality of sensors on the same fiber, either using multiplexing techniques or distributed sensing to reduce the spatial resolution to a few millimeters or less [7]. Thanks to these properties, FOS are making substantial progress in sensorizing miniature medical devices and are becoming a key technology in sensor-assisted medical insertions.

One of the main application scenarios for sensors on-board medical devices is represented by epidural anesthesia [9], which is gaining traction as a pain management method very common during labor [10]. The market for disposable devices for epidural anesthesia is expected to exceed $16 billion in 2027 [11], with a large share dedicated to the onboard sensing intelligence. Unlike some of the previously described biomedical applications, epidural insertion is performed with a hollow thin rigid needle with an 18 Gage thickness. This rules out many of the commercial sensors as their form factor does not fit the size of the needle itself.

Epidural anesthesia is a challenge for a clinician, as is involves inserting a percutaneous catheter that is terminated by a Tuohy tip (rotated by 90° from the needle axis) with a syringe that delivers the anesthetic fluid. The needle pierces the skin and travels through a series of tissues having different stiffnesses, before reaching the epidural location (2–6 mm thickness) between the ligamenta flava and the spinal cord. The two main methods for assisting epidural anesthesia are based on perceptive nonquantitative indicators [12]. The hanging drop method relies on the pressure differential on the epidural space, which causes a bubble of liquid to be sucked back into the needle. The loss of resistance relies on the clinician identifying the changes of pressure in the syringe as it travels through the hard ligamentum flavum tissue.

In order to provide quantitative and sensory based assistance to epidural anesthesia, methods based on Fiber Bragg Gratings (FBGs) have been proposed. Carotenuto et al. reported [13] and tested [2] a method based on an FBG mounted inside the needle that converts the tip force into a detectable strain. Similar work was recently proposed by De Tommasi et al. [14] using a single FBG assembled in the epidural needle. Ambastha et al. [15] also proposed an FBG-sensing method adapted to lumbar insertion and validated it on an animal model. These FBG sensing methods substantially translate the loss of resistance into a quantitative method as the sensor within the needle detects the force change.

The FBG methods have two limitations. At first, the FBG is mounted on the inner part of the needle, hence potentially obstructing, in part, the delivery of the anesthetic fluid. In addition, this method does not fully exploit the multipoint sensing capability of the optical fiber, providing a method that estimates whether the epidural space has been reached but without providing a guidance system that can support the clinician to actually reach the target region. Experimental data shown by FBG sensors [2] show a high variability between each output metric, as the differences in thickness of each layer, and the changes in the angle of insertion of the needle, have substantial impacts on the forces exerted on the needle.

In order to provide a full guidance system, a shape sensing method can be implemented [16]. Shape sensing methods make use of a single multicore fiber [17], or multiple fibers [18], to reconstruct in real time the deformation of the sensorized device without the use of radiation. Methods based on multicore fibers or multicore FBGs are used in flexible medical devices or soft manipulators [19], as they consistently detect distributed curvatures with narrow bending radii, by detecting the strain differential between multiple cores [20]. Multicore fibers are, however, unsuitable for rigid needles, as they cannot detect large bending radii, which are typically as high as thousands of meters in epidural needles since the displacement at the tip of the needle is in the order of fractions of a millimeter [21].

The approach introduced in [21] instead relies on multifiber distributed sensing and converting the local strain values into the needle shape. The system can work with three fibers [22], or four fibers for additional temperature compensation [23] and is well suited for rigid needles such as the epidural catheter. It relies on a multiplexed network of custom-made fibers, engineered to increase Rayleigh backscattering, which enables the scattering-level multiplexing arrangement [24].

In this work, we propose and experimentally assess a shape-sensing guidance system based on four simultaneously scanned distributed optical fiber sensors based on high-scattering nanoparticle-doped fibers. We evaluated the system over a phantom, which mimics the stiffness and sequence of layers of human tissues, and is in use for training clinicians for epidural insertion. We collected and classified the shape recorded for 25 handmade experiments of insertion, detecting in real time the shape and correlating it to the events occurring during the insertion. By estimating the silhouette of the needle, we were able to estimate how the tissues deformed the epidural device continuously through the process and without depending on the insertion speed and entry angle. We evaluated the sum of the bending angles and their temporal variations as key metrics for drawing a guidance system for anesthesia.

## 2. Materials and Methods

### 2.1. Concept of the Work

**Figure 1:**
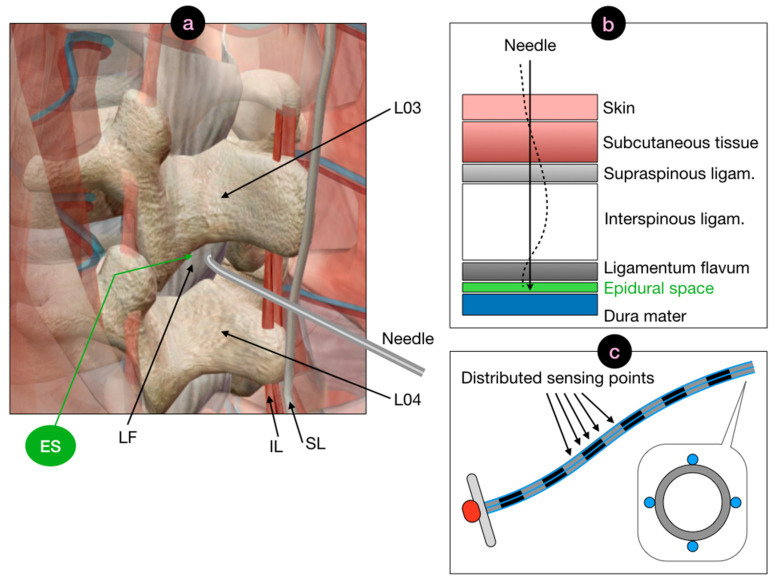
Rationale of the proposed method for guidance of the epidural anesthesia. (**a**) Anatomical view of the epidural space (ES), located behind the ligamentum flavum (LF) located between L03 and L04 vertebrae. The needle pierces the skin, crossing the subcutaneous fat layer, and progresses through layers of skeletal tissues such as supraspinous ligament (SL), interspinous ligaments (IL) located in proximity of the interspinales lumborum, and finally crosses the hard LF tissue. (**b**) Sequential view of the tissues penetrated by the epidural needle from the outer skin to the epidural space. As the needle progresses through tissues with different stiffness, it tends to bend into different patterns. The shape-sensing method reconstructs the needle silhouette and guides the insertion through the ES. (**c**) Overview of the method for shape sensing. Four fibers are mounted externally to the needle in the east, north, west, south cardinal points recording distributed strain patterns with millimetric spatial resolution.

The concept of the work is illustrated in Figure 1, which shows the anatomical view of the ES localization, its tissue sequence counterpart, and the shape sensing method. The ES is located after the ligamenta flava in the lumbar region, prior to the dura mater of the spinal cord. Currently, clinicians insert the epidural in the region located between L03 and L04 vertebrae. In order to position the needle in the ES, several layers of tissues have different stiffness and thickness are penetrated. At first, the needle pierces the skin with the underneath subcutaneous fat; then the supraspinous ligament, which is a hard tissue that can cause curvature in the epidural needle. The following interspinous ligament layer is soft and has a jelly-like consistency, which causes the needle to almost maintain its bending pattern. Finally, the ligamentum flavum tissue is reached, which is a hard skeletal tissue that can cause the needle to deflect, missing the ES.

Insertions of the epidural needle through the ES depend on the hand of the operator, the entry angle, and the specific consistency of each tissue, and can result in very different force patterns [14]. In general, insertions are either characterized by an almost straight pattern, quite insensitive to the sequence of tissues, or by a visible bending of the needle as it crosses the differential of stiffness between interspinous and supraspinous ligaments. Incorrect insertions are mostly characterized by a final deflection of the needle, which results in a lack of pressure rise for force-sensing methods [13] and are better rendered by a shape change observed towards the end of the experiment.

The method proposed in this work maintains the shape and form factor of the epidural needle including the diameter of the inner channel. The cross-section of the needle shows the four external sensors, each performing real-time simultaneous distributed sensing without the use of switches that would delay the data acquisition process.

### 2.2. Experimental Setup

**Figure 2:**
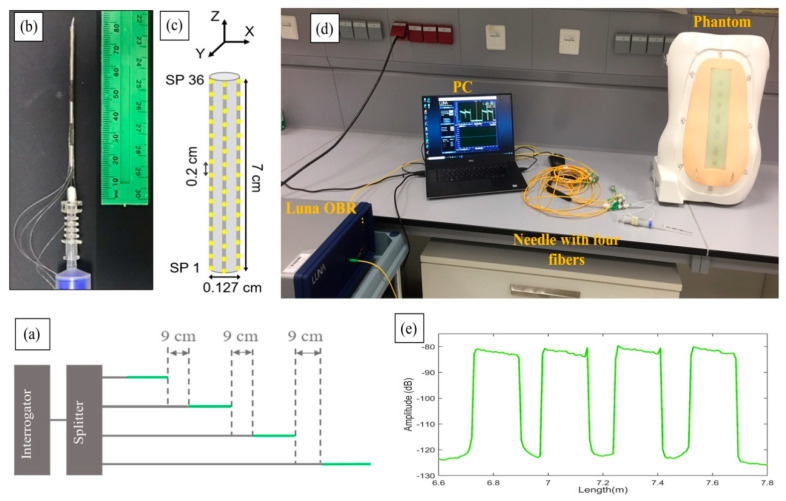
Experimental setup of the work. (**a**) Schematic of the multiplexing methodology of the NPDFs. (**b**) The upper view of the fiber mount on the needle. (**c**) The schematic of the fiber arrangement on the needle. (**d**) Photograph of the whole setup of the experiment. (**e**) Backscattering trace of the OBR in proximity of the 4-fiber sensing setup.

The experimental setup of the shape-sensing method is shown in Figure 2, which shows the analyzer and distribution network, the needle with the surrounding fibers and the phantom that allowed the test of the needle penetrations. The system is based on the optical backscatter reflectometer (OBR) method [25], using a network of high scattering fibers arranged in a scattering-level multiplexing (SLMux) network [23].

The setup shown in Figure 2d consists of a Luna OBR (Luna OBR 4600, Luna Inc., Roanoke, VA, USA), PC, phantom, 1 × 4 splitter, and epidural needle with four nanoparticles-doped fibers (NPDFs). The fibers were glued along the epidural needle as depicted in Figure 2b and connected to the Luna OBR interrogator via the splitter. The fiber fixing method used in this work, and the needle calibration method used to detect the strain, are analogous to [23].

NPDFs are formed by doping MgO nanoparticles into the core of single-mode fibers (SMFs), with the method described in [24]. The scattering level of NPDFs is 35–40 dB higher than the level of SMFs. This difference in scattering allows spatial multiplexing by having SMF fiber extenders at each channel of the splitter, which allow the scattering contributions of each NPDF not to overlap with each other. Figure 2a illustrates the scattering level multiplexing arrangement. The green lines are NPDFs and the grey lines are SMFs. The NPDFs are connected with SMF pigtails, so that each SMF is about 9 cm longer than the previous SMF+NPDF pair.

From the schematic diagram in Figure 2c, we see that the NPDFs are placed perpendicularly (at 90-degree angle) to each other along the needle (d = 0.127 cm), forming two opposite pairs, north-south and east-west. Since the OBR is used in distributed mode, the integration of NPDFs allows the needle region to have multiple sensors with a resolution of 2 mm. The length of the NPDFs region on the needle is approximately 7 cm, resulting in 36 sensing points (SPs) for each NPDF that are acquired by the OBR at 1 s speed. These values represent a good trade-off between acquisition speed, accuracy in strain sensing, and spatial resolution. Further processing and statistics were coded in Matlab (Mathworks, Natick, MA, USA).

The epidural needle (Balton, Warsaw, Poland) used in the experiments has a Tuohy-shaped silhouette with curved tip, 18 Gage diameter, and diameter of the inner hole of ~0.8 mm.

The phantom (P61, 3B Scientific, Hamburg, Germany) replicates the anatomy of the human spine and the stiffness and thickness of each of the layers encountered during epidural insertion displayed in Figure 1. The phantom is used to train clinicians on the loss-of-resistance method [12], and therefore the results obtained in this work can be compared to the performance obtained in training tools for anesthesiology.

### 2.3. Needle Insertions

To replicate the insertion of the needle to the epidural space, several experiments were performed covering different cases and methodologies. Experiments were performed by manually inserting the needle into the phantom location, attempting to reach the epidural space layer. Empirically, we categorized four types of experiments, classified as follows:

- Straight insertions: the needle is inserted with a strong movement that forces the needle through the sequence of layers without allowing it to bend in a significant way.
- Large curvature: the operator inserts the needle with a weak motion, gently pushing the needle all the way through the tissues. This results in a larger bending in correspondence of the transition between the stiff skeletal tissues and the jelly-like interspinous ligament.
- Reduced bending: the needle curves gently during the insertion, and the operator attempts to straighten its form in order to penetrate the harder tissues.
- Failed insertions: the needle suffers from a high deflection which causes the operator to miss the epidural space.

### 2.4. Shape Reconstruction

**Figure 3:**
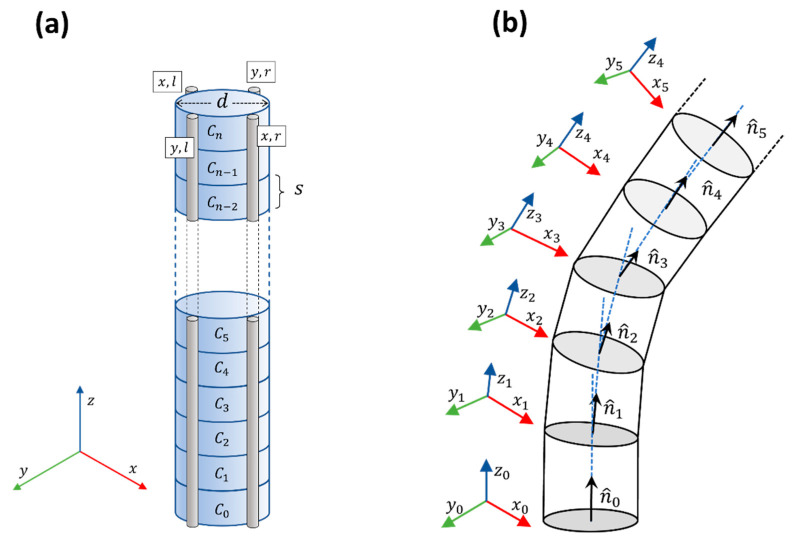
(**a**) Disposition of the fibers fixed along the length of the needle. Two fibers, tagged as x,l and x,r lie on the plan xz, while two fibers, tagged as y,l and y,r lie on the plan yz; (**b**) fiber bending is calculated as an incremental rotation of the normal vector ${\hat{n}}_{i}$ in every fiber sector defined as a needle portion between two sensing points.

The shape reconstruction of the needle was achieved by equipping the device with four fiber sensors, arranged in a 90° angle configuration, as shown in Figure 3a. In this regard, we split each section C_i_ between two consecutive sensing points on the *xyz* space (*z* = fiber axis; *xy* = cross-section plane). The idea of this configuration is to measure the bending-induced distributed strain along the needle on two perpendicular coordinates characterized by the presence of two coupled fiber sensors, facing each other. The use of four fiber presents advantages, in particular, improved accuracy of the strain detection and the possibility to compensate the temperature variation along the length of the needle. Moreover, the 90° configuration can be matched with a simplified and efficient 3D shape reconstruction algorithm that is particularly good for devices that present small bend angles [26].

To explain the features of the reconstruction algorithm adopted in this work, it is necessary to remember that with help of the distributed sensing and the OBR interrogator it is possible to select the number n of sensing points along the length of the needle. The preliminary assumptions that validate the algorithm are the following:

- The needle is rigid enough to avoid deformation and torsion.
- The base of the needle is not rotated during the insertion.
- The bending angle θ is small, so that sinθ≅tanθ≅θ.

It is now possible to model the needle as a stack of n small cylinders, with diameter d and height s, equivalent to the distance between two neighbor sensing points. With a straight needle, the normal vector ${\hat{n}}_{i}$ of each cylinder is directed along z while the two couples of coordinated fibers lie on xz-plane and yz-plane, respectively. Due to bending, each couple of coordinated fibers detect the strains $S_{x}$ and $S_{y}$. In a normal condition the opposite fibers measure opposite strains. An occurring temperature gradient can be removed since it affects all the fibers [23]. The strain can be transformed in a rotation of the normal vector ${\hat{n}}_{i}$.In particular, at every section the normal vector is rotated by the following angles:

(1) $$\theta_{x,i+1}=\tan^{-1}\left[\left(S_{x,i,l}-S_{x,i,r}\right)\frac{s}{d}\right]$$

(2) $$\theta_{y,i+1}=\tan^{-1}\left[\left(S_{y,i,l}-S_{y,i,r}\right)\frac{s}{d}\right]$$

These rotations represent the rotation of the reference frame, so that the couples of opposite fibers always lie on the relative $x_{i}z_{i}$ and $y_{i}z_{i}$ planes, as shown in Figure 3b. Section by section, it is possible to reconstruct the direction of the normal vector and consequently the needle 3D shape. Exploiting the assumption of small bending, the approximation becomes linear, easy to implement and compute. This operation has specific bio-medical applications, such as micro-invasive robotic surgery, where minimal deformation must be measured and reconstructed.

### 2.5. Bending Metrics for Assessment

**Figure 4:**
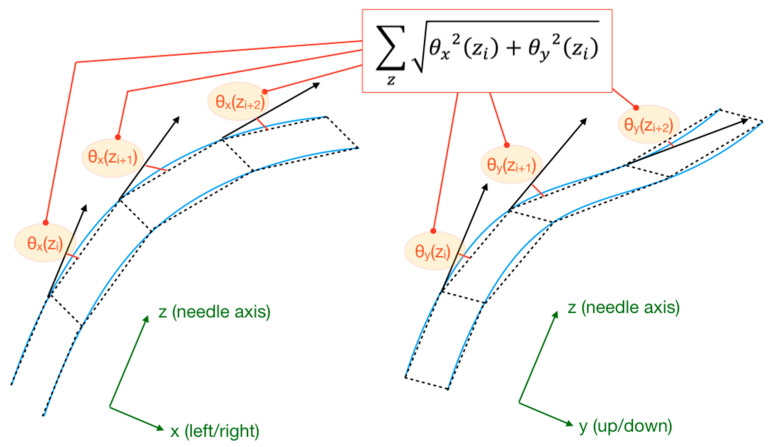
Geometrical visualization of the sum of the projected bending angles (SPBA), which is the metric used to evaluate the bending pattern for each experiment. The figure shows how each local bending angle contributes to the SBPA calculation for the needle section under investigation.

The assessment of the shapes of the needle observed through the experiments was translated into a metric that allows evaluation of the bending pattern observed in the experiments. We used a metric that we define as the sum of the projected bending angles (SPBA), labelled *B*, which is geometrically represented in Figure 4.

The SBPA is calculated by projecting each angle that measures the local bending along the needle axis *z*, both along the horizontal axis (*x*, left-right) and along the vertical axis (*y*, up-down). For each location *z_i_*, the angles *θ_x_*(*z_i_*) and *θ_y_*(*z_i_*) are calculated; then, their projection is computed as [*θ_x_^2^*(*z_i_*) + *θ_y_^2^*(*z_i_*)]^−1/2^, under the small-angle condition tan(*θ*) ≈ *θ* [21]. Then, the projected bending angles are summer along a section or the whole needle, from the location *L*_1_ to *L*_2_:

(3) $$B=\overset{L_{2}}{\underset{i=L_{1}}{\sum }}\sqrt{\theta_{x}{}^{2}\left(z_{i}\right)+\theta_{y}{}^{2}\left(z_{i}\right)}$$

The calculation of *B*(*t*) along the time coordinate *t* provides a quantitative metric of the bending pattern observed in each experiment. Insertions that return a low value of *B* imply that the needle maintains its straight form. High values of *B* are observed for curvatures that apply to the whole section of the needle. On the other hand, sudden changes of *B* are observed when the epidural device crosses stiff portions of the tissues, or when the needle straightens after bending upon piercing a hard layer.

Since epidural needles are bent all the way through the whole section [21], the metric *B*(*t*) yields similar values for nearly any choice of the interval (*L*_1_ ÷ *L*_2_). In the following section, we apply Equation (3) to 10 datapoints distributed for a 25 mm section of the needle, located at 17.5 mm from the tip. This region is similar to the tip-to-sensor distance in some epidural sensor positioning [13], and also corresponds to the region that is exposed to the bending as the needle crosses the skeletal tissue of the ligamentum flavum. In addition, by choosing a portion of the needle that is intermediate between the tip and the distal side, we can ensure that the investigated portion of the needle is located within the region that experiences the tighter bending, while leaving regions that are external to the phantom out of the analysis.

In addition to *B*, we also track the rate of change of the SBPA, i.e., ∂*B*/∂*t*, which returns an indicator of the dynamic change of the bending patterns. This allows distinguishing between a rapid increase of curvature and the events causing the needle to straighten after being subjected to substantial bending.

To compare different experiments we normalized the time basis, since all experiments had different durations depending on advancement speed set by the operators and their manual operations. The normalized time ranged from 0 (reference, calculated at the start of the insertion) to 1 (end of the insertion). Different SBPA patterns were plotted on the same normalized time axis by dividing each time axis by the experiment duration and applying a spline fit to each SBPA in post processing.

## 3. Results

### 3.1. Needle Shape Estimation

The 3D shape sensing system allows a real-time reconstruction of the epidural needle silhouette, visualized in correspondence to each scan of the OBR instrument. The 25 insertion experiments were divided into each class and grouped into videos in order to find common patterns and analyze the bending configurations through SPBA analysis.

**Figure 5:**
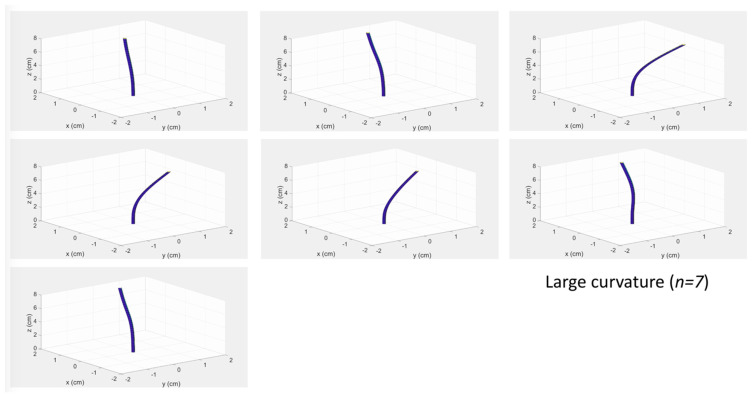
Screenshot of a video reconstructing the 3D shape of the epidural device through the penetration to the epidural space within the phantom. The videos for each experiment group are shown as Supplementary Materials.

The videos displaying the 25 reconstructed shapes are enclosed as Supplementary Materials, grouped into the four classes of experiments (four files). A screenshot of the video showing the experiments having large curvature is displayed in Figure 5. The 3D-reconstructed shape shows the profile of the needle reconstructed only through the optical fiber sensors.

### 3.2. Analysis of Bending Patterns

In the following, we analyze bending patterns observed by the epidural needle as reconstructed in the three-dimensional space by the shape-sensing algorithm, and the corresponding SPBA values. In order to facilitate the visualization of the silhouette of the epidural device, the shapes were presented in a rescaled chart. The needle axis (*z*) is presented over 0–8 cm corresponding to the length of the needle, while the cross-section (*xy*) is expanded for a better display of the curvatures; the 1.2 mm scalebar, corresponding to the diameter of the needle, is reported on each chart. We analyzed the cinematic of the insertion by reporting the shape reconstructed from the reference condition (needle out of the phantom) recorded at 0 s until the end of experiment.

**Figure 6:**
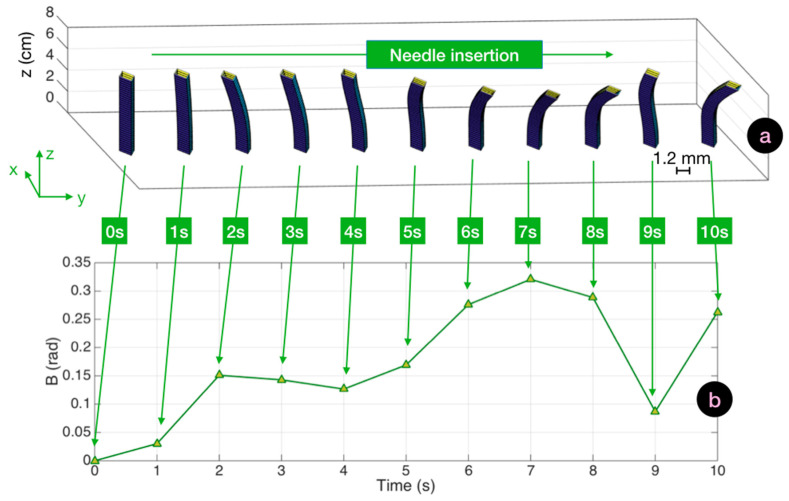
Bending cinematic for a straight insertion and the corresponding SBPA chart. (**a**) Cinematic of the straight insertion (10 s duration), displaying the reconstructed silhouette of the needle (1.2 mm cross-section on *xy*, 8 cm length along *z*) through the whole insertion. (**b**) Corresponding SBPA chart showing the value of *B* corresponding to each bending pattern.

In a straight insertion, the needle maintained a shape with a shallow curvature throughout the insertion. A bending differential could be observed when the device passed through the intermediate layers without a strong deformation of its profile. This case scenario is analyzed in Figure 6, which displays the cinematics of the reconstructed three-dimensional shapes and their corresponding *B*(*t*) traces. From the initial insertion, the needle bent leftwards, and progressed with a curvature leaning towards southeast direction to the target. The values of *B* increased up to 0.3 rad to reach a maximum after 7 s. Maximum vertical bending was observed at 7 s at 0.04 rad (2.3°) in the central point, while the average bending through the whole needle length was 1.2° at the point of maximum SPBA. The decrease in SBPA recorded at 9 s could be caused by the operator pushing the needle to a straighter shape in an effort to cross the hard compound of tissue.

**Figure 7:**
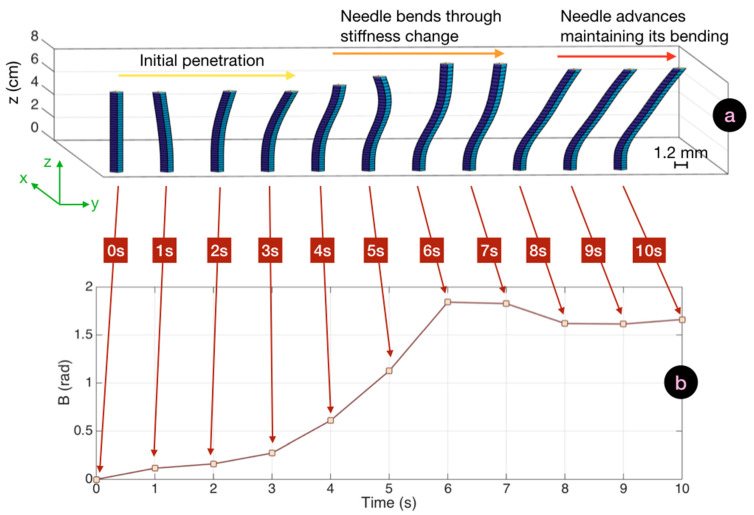
Bending cinematic of an insertion with large curvature and the corresponding SBPA chart. (**a**) Cinematic of a straight insertion (10 s duration) displaying the reconstructed silhouette of the needle (1.2 mm cross-section on *xy*, 8 cm length along *z*) through the whole insertion. (**b**) Corresponding SBPA chart showing the value of *B* corresponding to each bending pattern.

In Figure 7 we report the bending pattern observed for an insertion characterized by a large curvature. The initial pattern was similar to the previous experiment. Up to 3 s; the values of *B* were less than 0.3 during this time, and the needle appeared to be gently bending towards the right direction. At this point, curvature became evident, as the operator struggled to pass through the first hard/soft interface at the boundary of the supraspinous ligament. This resulted in a steep increase of *B* which reached a 1.8 rad value, and in this condition the maximum local bending observed at 6 s corresponded to 4.3°. After this phase, the needle advanced to the target without further increase of the bending pattern. The needle rotated further towards the right side, with a motion similar to the previous experiment, but maintaining its directionality and shape.

**Figure 8:**
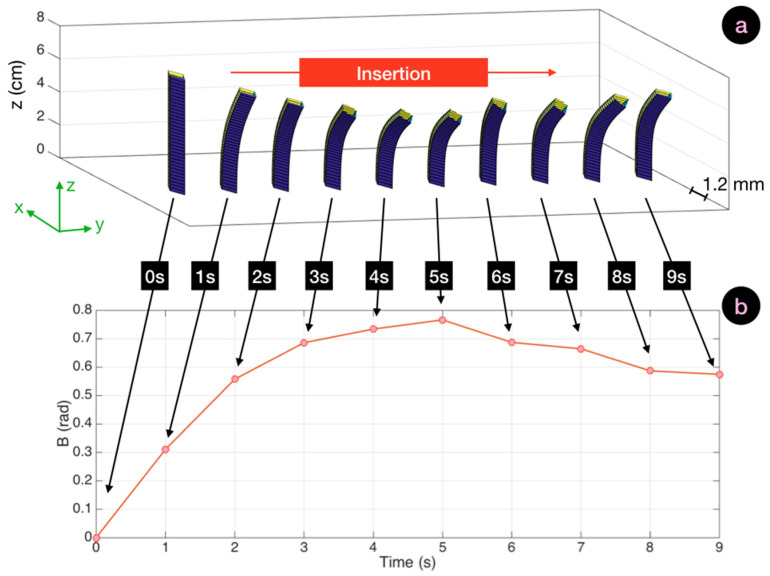
Bending cinematic for an insertion with reduced bending, and the corresponding SBPA chart. (**a**) Cinematic of a straight insertion (9 s duration), displaying the reconstructed silhouette of the needle (1.2 mm cross-section on *xy*, 8 cm length along *z*) through the whole insertion. (**b**) Corresponding SBPA chart showing the value of *B* corresponding to each bending pattern.

In Figure 8, we display the bending pattern observed with reduced bending. From the initial entry point, the needle bent towards the right direction and continued to further curve for the first 5 s, reaching 0.76 rad as peak *B* value, a further increase from the straight insertion. The rise of *B* was progressive until the needle reached its maximum bending pattern. Then, as the operator advanced the epidural device through the soft inner layers of tissue, the needle straightened and only the tip of the needle remained significantly bent. This corresponded to a 23% reduction of *B*, until 0.59 rad towards the end of the experiment.

**Figure 9:**
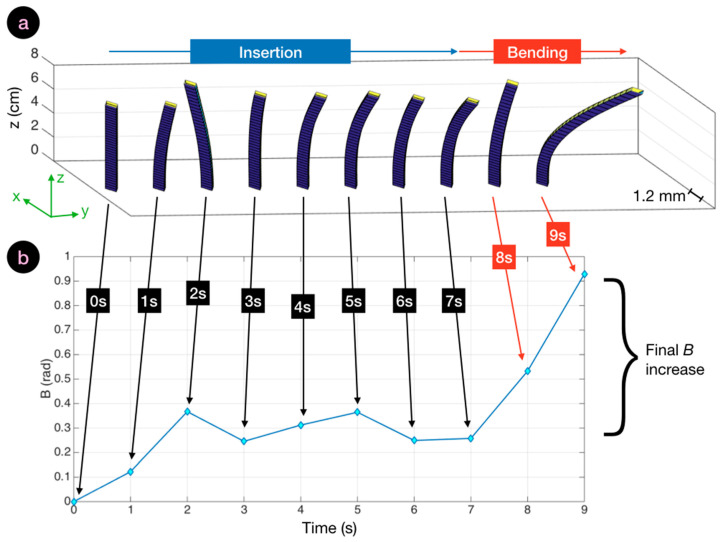
Bending cinematic for a failed insertion and corresponding SBPA chart. (**a**) Cinematic of the straight insertion (9 s duration), displaying the reconstructed silhouette of the needle (1.2 mm cross-section on *xy*, 8 cm length along *z*) through the whole insertion. (**b**) Corresponding SBPA chart showing the value of *B* corresponding to each bending pattern.

Finally, we show in Figure 9a cinematic of the epidural needle silhouette observed for a failed insertion in which the needle was deflected by the operator in the final layers of tissues. For the first 7 s, the needle advanced into the tissue with a pattern similar to Figure 6, as the shape of the needle was relatively straight, with a torsion recorded around 2 s, but immediately corrected into the next frame. At 8 s, the needle abruptly bent, and after 9 s *B* rose to the final value of 0.91 rad. This was caused by the rightwards torsion of the needle, that is observed in the final frame of the cinematic view.

Through shape sensing, we can identify the differences in the bending patterns caused by the different insertion motions. The anomalous pattern observed in Figure 7, for bending with a tight curvature, shows that the needle was bent all the way through, with the maximum curvature observed at the distal end. In the failed insertion of Figure 9, the needle curved along its entire structure, with the maximum angle observed in the middle point where the needle silhouette has an arc-like shape across the final frames of the experiment. In the other scenarios, where the curvature was lower, the bend of the needle was evident only in the tip, as the firm hand of the operator prevented the needle from twisting in the distal section.

### 3.3. Assessment of Bending Metrics

**Figure 10:**
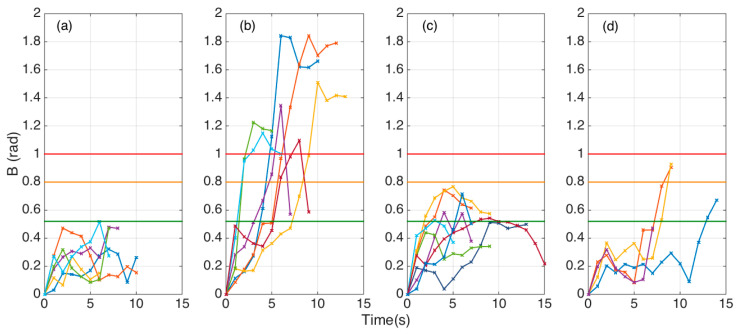
SPBA temporal patterns for all epidural insertions into the phantom. The charts shows the variations of *B* over the whole experiment duration for 25 insertions, divided into each class. Each *B*(*t*) trace is represented with a different color. The vertical axis shows the threshold levels for determining each type of insertion: *B* = 0.52 (green), *B* = 0.8 (orange), *B* = 1.0 (red). (**a**) Experiments showing a straight insertion (*n* = 6). (**b**) Insertions showing a tight needle curvature that progresses through the target epidural space (*n* = 7). (**c**) Hard penetrations showing bending that straightens through the insertions (*n* = 8). (**d**) Failed insertions characterized by a strong deflection towards reaching the target zone (*n* = 4).

Analysis of the SPBA patterns allowed comparisons of epidural insertions, and served as the guidance system for the operator. The *B*(*t*) traces for all 25 experiments, divided into each category as in Section 2.3, is shown in Figure 10.

Straight insertions (*n* = 6) show a value of *B* that ranged between 0.1 and 0.5 for most of the insertion duration. Each experiment, due to the variations of the forces exerted by the operator, yielded a slightly different pattern. However we can observe that in all the experiments the SPBA did not exceed the value of 0.52. Considering that *B* is the sum of 10 datapoints, the average local bending did not exceed 0.052 rad = 2.9°.

Large curvature experiments (*n* = 7) showed a completely different trend, as the SBPA largely exceeded the value of 1, approaching 1.8 for two experiments, halfway through the insertion. As these experiments were made with a weaker motion that allowed the needle to bend tighter as it crossed the hard skeletal layers, we observe SBPA values that ranged between 1.1 and 1.8 in the maximum location. After reaching the maximum, the insertion maintained or slightly decreased the bending.

Reduced bending experiments (*n* = 8) showed that *B* always overcame the 0.52 early or halfway through the insertion, but did not achieve a target value of 0.8. After approaching this value, the bending decreased as the operator straightened the needle pulling through the tissues.

Failed insertions (*n* = 4) had a different pattern, as the needle proceeded with small bending (*B* within 0.1–0.4), but showed an abrupt increase towards the end (with an increase of *B* approximately equal to 0.5).

**Figure 11:**
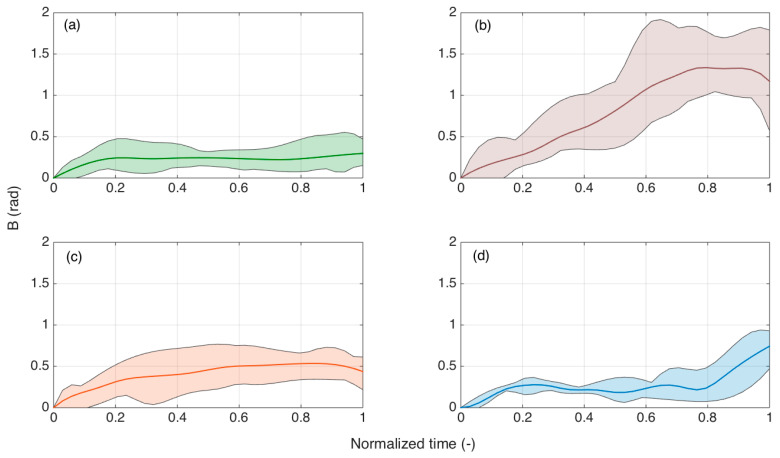
SPBA patterns reported over normalized time (0 = start of the insertion; 1 = end of the insertion). Solid lines show the mean *B* value for each normalized time reported for all the experiments showing a similar pattern to Figure 10. Shaded regions show the minimum and maximum values among all the experiments. (**a**) Straight insertion; (**b**) large curvature; (**c**) reduced bending; (**d**) failed insertions.

Figure 11 shows the bending patterns *B* as a function of the normalized time. This allows a comparison within experiments that have different durations, as the insertion speed for manual insertion varied. In the charts, we show the range of maximum and minimum values for each *B*, and average values among experiments of the same class.

Here we can observe the different patterns that occur for each type of experiment. Straight insertions had a low *B* value through the insertion. On the other hand, e experiments that showed large bending tended to overcome *B* > 1 at 40–80% of the experiment duration, with curvature that appeared to decrease in the last 20% of the insertion process. Conversely, the third type of insertion showed bending that progressively increased and decreased towards the end of the insertion. The pattern of failed insertions was different, as the bending was always low for the initial 80% of the experiment, but rapidly increased in the last 20%.

**Figure 12:**
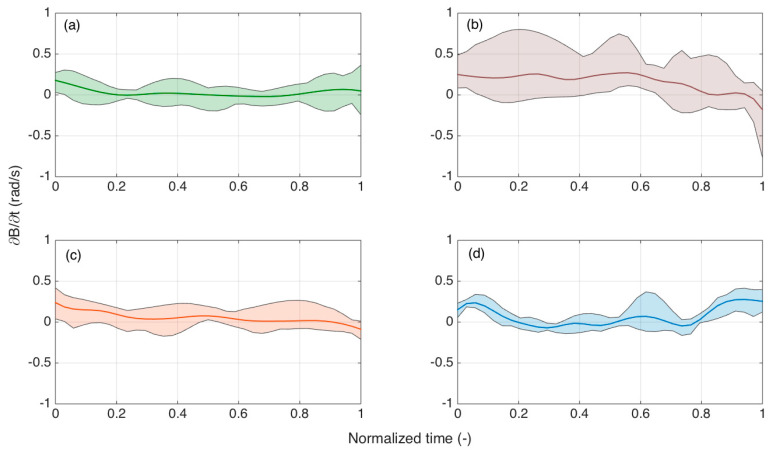
Analysis of the time derivative of SPBA over normalized time for each class of experiments. The charts show ∂*B*/∂*t* patterns, solid lines = mean for all experiments, shaded regions = minima/maxima at each normalized time. (**a**) Straight insertion; (**b**) large curvature; (**c**) Reduced bending; (**d**) failed insertions.

Further analysis was conducted by analyzing the time derivatives of *B*, as reproduced in Figure 12, as a function of normalized time, and grouping the different experiments into shaded plots. As the OBR acquired data with 1 s speed, the calculations of ∂*B*/∂*t* had an accuracy inferior to the computation of *B*, but could provide a trend that diversified the failed insertions from those that correctly reach the ES.

We focused, in particular, on the final parts of the experiments for normalized time >80%. For all first three bending patterns, the derivative was either slightly positive or negative, showing that the curvature was mostly decreasing. For all failed experiments, instead, the derivative appeared to increase from normalized time of 80% to 95%. This increase of ∂*B*/∂*t* can be used as a distinguishable metric to recognize insertion failures.

**Figure 13:**
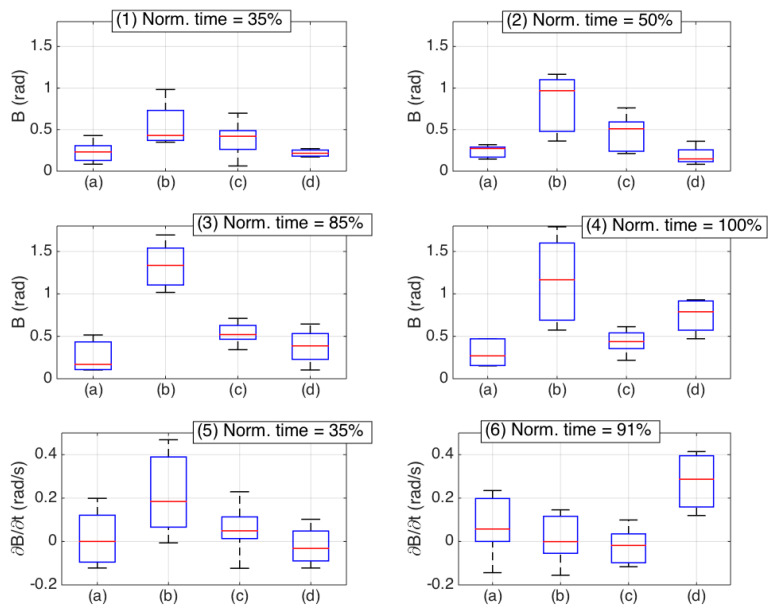
Box plots showing the most important metrics for epidural insertion classification. Each chart shows a different metric obtained for *B* or ∂*B*/∂*t* at a different normalized time. (**a**) Straight insertion; (**b**) large curvature; (**c**) reduced bending; (**d**) failed insertions. Box plots show median value (red), 25th and 75th percentiles (blue box), minimum and maximum values (black error bars). No outliers were identified. (**1**) *B* values for normalized time = 35%; (**2**) *B* values for normalized time = 50%; (**3**) *B* values for normalized time = 85%; (**4**) *B* values for normalized time = 100%; (**5**) ∂*B*/∂*t* values for normalized time = 35%; (**6**) ∂*B*/∂*t* values for normalized time = 91%.

Figure 13 shows some of the main metrics of the bending patterns as a statistical box plot [27]. Here, we compare different *B* and ∂*B*/∂*t* values for different normalized times and propose a set of six different metrics that can allow users to perform identification of the epidural insertion quality, and perform classification tasks. The first and fifth chart show the values obtained after 35% of the insertion process. Both metrics allow a solid detection of the insertions having large curvatures, as the values observed for large curvatures largely exceed the other insertions. A similar consideration, with an ever higher distinguishable pattern, can be performed by analyzing, in the second and third chart, the mid-point patterns (normalized time 50% and 85%, respectively) of *B*. This is highest for the large curvatures, followed by reduced bending, and has similar values for straight and failed insertions. Late metrics are shown in the fourth and sixth charts, reporting respectively *B* at the end of the insertion and ∂*B*/∂*t* at 91% normalized time. Here, we can see that the discrimination between straight and failed insertions is the most successful, as the values observed for failed insertions are always the highest. The results presented in this statistical analysis are based on a dataset of 25 experiments. However, they provide a background for quantitative and qualitative evaluation of bending patterns during epidural insertion, and draw some timestamp-related metrics that allow development of future classification methods.

## 4. Discussion

Epidural anesthesia is characterized by a high variability of results, as the layers encountered by the needle as it progresses from the outer skin to the ES vary in thickness and stiffness and, therefore, different patterns are observed [14]. So far, perceptive methods (loss of resistance, hanging drop) are in use to support clinicians in the task [12]. FBG-based methods provide quantitative evaluation of the force exerted on the needle tip and can replace the perceptive methods with a quantifiable counterpart, which corresponds to the FBG wavelength shift observed during the insertion [13].

The method we propose takes full advantage of distributed sensing on one side, and of shape sensing on the other side. By using distributed sensing [7], setting the OBR at the best trade-off between speed of acquisition, strain accuracy, and gage length [25], it is possible to turn the whole fiber into a sensor, and achieve dense mapping of the surface of the needle for shape reconstruction. In this work, shapes were estimated using 144 sensing points on the needle distributed over the outer cylindrical surface of the needle (359 mm^2^), which implies that each set of four sensing points (considering three to map the shape, and an additional one to compensate temperature [23]) maps 12 mm^2^ of needle surface. This narrow spatial resolution allows very detailed mapping of the shape and is returned as real-time feedback.

In this work, we aimed for the first time at using the whole shape data, and in particular the patterns derived from the total bending of the needle, as means to monitor the status of the insertion. We showed in the previous section that by acting on different metrics and features, we can discriminate different events occurring during insertion without having to place any sensor within the wall of the epidural device, and therefore leaving untouched its actual capability to deliver the anesthetic fluid once the ES has been reached.

This method can be considered as both complementary and alternative to other tracking systems. On one side, loss of resistance and hanging drop methods are simple and effective, but rely entirely on the clinician’s capabilities. The method based on shape sensing can, therefore, provide the additional guidance system that can be useful to effectively reach the ES. Here, the shape sensing method can serve the role of an augmented reality system for the surgical device [28], as it combines the perceptive information with an auxiliary real-time tool. A similar consideration can be drawn for FBG-based methods, as they aim to provide an identification of the ES but do not embed the guidance system required to assist the whole insertion. For these reasons, the proposed shape sensing device is intended to complement these methods by adding the guidance function, and clearly the real-time visualization of the needle silhouette serves as valuable assistance and potentially as a training method for clinicians [29].

The shape-sensing method can be used to identify the ES and guide the penetration of the needle to the ES without relying on any other additional methods or functions. The results of this work, and the analysis of the bending patterns, show that even when used alone, there are metrics that discriminate different events occurring during insertions. Some events occur half-way through the process (such as the identification of large curvatures), while others represent indicators that act more as failure identifiers, such as the detection of a bending increase during the late stages of the insertion. This occurrence is in agreement with the FBG-based system proposed by Carotenuto et al. [13], as the large needle deflection that causes the operator to miss the ES is visualized through the FBG sensor as a lack of a pressure peak at the end of the needle penetration. Further research should support the advancement of the proposed sensing method into a medical device, proving that the same consistency of results observed in Section 3 can be achieved when performing in vivo experiments. The availability of machine learning based classification systems, very common in the analysis of large experimental datasets to extract features and classify events and recently successfully extended to surgical devices [30], provides an additional opportunity for translational purposes in this direction.

The results obtained in the present work are in agreement with prior shape-sensing setups, which show an agreement in the order of ~1% between the reference (ground truth) and the proposed method [31] obtained for shape sensing in a curvature setup outside of a phantom, and with [23] in a temperature-varying phantom.

## 5. Conclusions

We assessed a 3D shape-sensing system for needle guidance in epidural anesthesia. The system is based on a distributed shape sensing network arranged as a scattering-level multiplex system through the use of high-scattering, nanoparticle-core-doped fibers, and implemented real-time sensing through OBR. We assessed the system over 25 insertions on a phantom for epidural insertion, performing experiments that reproduced different conditions. Besides the accurate estimation of the needle silhouette, we identified bending patterns for each experiment by measuring the sum of the bending angles and its temporal derivative. This serves as key metric that can support the operator not only to determine whether the epidural space has been reached, but also to provide a guidance system to determine whether needle curvature is consistent with the correct penetration of the different types of tissue.

Future work will involve fusing three-dimensional shape data with an external camera system that estimates the input angle, to refer the shape to the surface of the external skin. In addition, it is envisioned that the use of machine-learning based methods may extract and analyze additional bending features for a more advanced classification.

## Data Availability

Data presented in this work are not publicly available at this time. Raw data and/or the reconstructed shapes maybe obtained upon reasonable request from the authors.

## Supplementary Materials

The following are available online at https://www.mdpi.com/article/10.3390/bios11110446/s1. Video S1: reconstructed 3D shape for straight epidural insertions (*n* = 6 experiments). Video S2: reconstructed 3D shape for insertions having large curvature (*n* = 7 experiments). Video S3: reconstructed 3D shape for insertions having reduced bending (*n* = 8 experiments). Video S4: reconstructed 3D shape for failed epidural insertions (*n* = 4 experiments).
